# The Synergy of Ciprofloxacin and Carvedilol against *Staphylococcus aureus*–Prospects of a New Treatment Strategy?

**DOI:** 10.3390/molecules24224104

**Published:** 2019-11-14

**Authors:** Katarzyna Zawadzka, Marta Nowak, Ireneusz Piwoński, Katarzyna Lisowska

**Affiliations:** 1 Department of Industrial Microbiology and Biotechnology, Faculty of Biology and Environmental Protection, University of Lodz, 12/16 Banacha Street, 90-237 Lodz, Poland; marta.nowak1@unilodz.eu; 2 Department of Materials Technology and Chemistry, Faculty of Chemistry, University of Lodz, 163 Pomorska Street, 90-236 Lodz, Poland; ireneusz.piwonski@chemia.uni.lodz.pl

**Keywords:** ciprofloxacin, carvedilol, antimicrobial synergy, *S. aureus*, β-blocker

## Abstract

*Staphylococcus aureus* infections are common and difficult to treat. The increasing number of drug-resistant staphylococcal infections has created the need to develop new strategies for the treatment of these infections. The synergistic antimicrobial activity of different pharmaceuticals seems to be an interesting alternative. The aim of this study was to assess the synergistic activity of ciprofloxacin and carvedilol against *S. aureus* strains. The antibacterial potential of ciprofloxacin and carvedilol was evaluated according to the CLSI guidelines. The calcium content in *S. aureus* cells was measured using flow cytometry and atomic absorption spectroscopy. Moreover, confocal and scanning electron microscopy were used to determine the mechanism of antibacterial synergy of ciprofloxacin and carvedilol. The antibacterial effect of ciprofloxacin was higher in the presence of carvedilol than in *S. aureus* cultures containing the antibiotic only. A significant increase in *S. aureus* membrane permeability was also observed. The simultaneous administration of the tested compounds caused damage to *S. aureus* cells visualized by SEM. Enhancement of the antimicrobial action of ciprofloxacin by carvedilol was correlated with an increase in free calcium content in *S. aureus* cells, morphological changes to the cells, and a reduction in the ability to form bacterial aggregates.

## 1. Introduction

*Staphylococcus aureus* is one of the most common human commensal microorganisms; however, it causes numerous opportunistic infections. *S. aureus* possesses an ability to evade the response of the immune system and can colonize many niches within the host organism [1,2,3]. In addition, it is a pathogen causing bacteremia and infections such as in the endocardium, skin and soft tissue, or in osteoarticular systems [3]. The wide range of *S. aureus* virulence factors and the spread of antibiotic resistance of the bacteria make these infections difficult to treat. *S. aureus* is responsible for infection in approximately 1% of all patients admitted to hospitals in the United States, and the cost of their treatment is estimated at 9.5 billion dollars per year [1]. 

A serious problem for healthcare is the development of drug resistance in staphylococci. For example, *S. aureus* strains resistant to penicillin were isolated shortly after the introduction of the drug. The bacteria produce enzymes, such as β-lactamase, which can hydrolyze bonds in antibiotic structures and reduce their antibacterial activity. The chemical modifications of naturally occurring penicillin have been of little avail because the resistance of *S. aureus* strains toward methicillin or oxacillin is frequently noted [4]. An increasing frequency of infection caused by methicillin-resistant *S. aureus* strains in recent years is especially worrying [3].

One of the main problems in modern medicine is expanding bacterial resistance to conventional antimicrobial agents and hence a reduction in the effectiveness of infectious disease treatment [5]. However, the search for/the development of new antimicrobial drugs can be a solution to this problem. Chauhan et al. [5] demonstrated strong antibacterial activities of novel benzimidazole derivatives against *S. aureus*. Gatadi et al. [6] synthesized new benzamide and 4-oxoquinazolin-3(4H)-yl)benzoic acid derivatives, which showed inhibitory activity against *S. aureus* as well. By contrast, derivatives of 4(3H)-quinazolinones and 1,2,3-triazole possess antimicrobial potential against multidrug-resistant *S. aureus* strains [7]. The simultaneous application of different antibiotics can be also an effective approach in the treatment of bacterial infections. Moreover, combination therapy is proposed as a potential strategy to fight drug-resistant bacteria [8]. 

An alternative to combination therapy with various antibiotics seems to be the use of the antimicrobial potential of non-antibiotic drugs [9]. Mazumdar et al. [10] studied ten cardiovascular drugs in terms of their antimicrobial activity and the highest potency was shown by oxyfedrine.

In our previous report, the antibacterial activity of high concentrations of carvedilol against Gram-positive and Gram-negative bacteria was described [9]. Carvedilol is a nonselective beta-adrenergic receptor blocking agent that also shows antioxidant and Ca^2+^ channel blocking activity [9]. Here, we report on the synergistic effect of ciprofloxacin and carvedilol against *S. aureus* strains including MRSA. Furthermore, the results of SEM and confocal imaging as well as calcium ion content in bacterial cells and biofilm formation were evaluated to explain the mechanism of antimicrobial synergy between ciprofloxacin and carvedilol.

## 2. Results

### 2.1. Antibacterial Potency of Ciprofloxacin and Carvedilol

**Figure 1:**
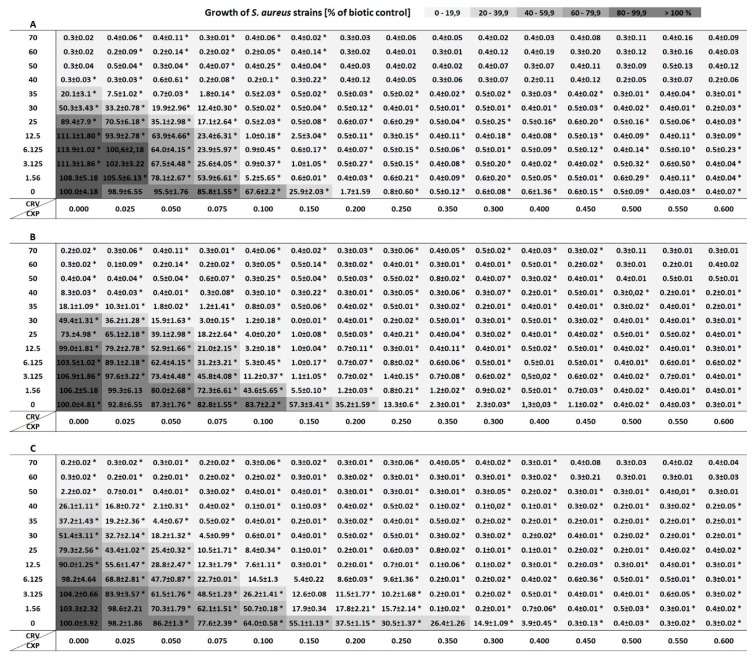
The growth of *Staphylococcus aureus* ATCC 6538 (**A**), ATCC 29213 (**B**), and ATCC 43300 (**C**) strains incubated with the addition of ciprofloxacin (0–0.6 mg/L) and/or carvedilol (0–70 mg/L). Data are shown as means of bacterial growth (percentage of biotic control) ± standard deviation (SD). Obtained results were analyzed using the one-way and two-way analysis of variance (ANOVA) with * *p* < 0.05.

In this study, the antibacterial synergy of ciprofloxacin and carvedilol against *S. aureus* strains was evaluated for the first time. The antibacterial potential of the antibiotic and the β-blocker against two methicillin-sensitive strains (ATCC 6538 and ATCC 29213) and one methicillin-resistant strain (ATCC 43300) is presented in Figure 1A–C. *S. aureus* strains showed weak susceptibility to ciprofloxacin at the concentration 0.025–0.1 mg/L. Addition of the antibiotic at the concentration of 0.1 mg/L caused a 33%, 17%, and 26% growth reduction of ATCC 6538, ATCC 29213, and ATCC 43300 strains, respectively. The minimum inhibitory concentration (MIC) values of ciprofloxacin against ATCC 6538, ATCC 29213, and ATCC 43300 strains were 0.25, 0.40, and 0.45 mg/L, respectively (Table 1). Treatment of the bacterial cells with carvedilol at the low concentration 1.56-6.25 mg/L did not inhibit *S. aureus* growth and even its slight stimulation was observed, while co-administration of ciprofloxacin and carvedilol led to the enhancement of bacterial growth reduction. For carvedilol, MICs were 40, 50, and 60 mg/L toward ATCC 6538, ATCC 29213, and ATCC 43300 strains, respectively. The growth of *S. aureus*, which was simultaneously treated with the antibiotic and β-blocker, was statistically significantly more inhibited compared with the bacteria incubated separately with the two agents. In the cultures supplemented with 0.1 mg/L ciprofloxacin and 1.56 mg/L carvedilol, the growth limitation of ATCC 6538, ATCC 29213, and ATCC 43300 strains was 95%, 56%, and 49%, respectively. The antibacterial effect of ciprofloxacin was from 1.3 to 13 times higher in the presence of carvedilol than in *S. aureus* cultures containing only the antibiotic. 

The fractional inhibitory concentration indexes (FICIs) obtained for the tested strains after incubation with the addition of the antibiotic and the β-blocker ranged from 0.081 to 0.139 and confirmed the synergistic effect of both pharmaceuticals (Table 1). To conclude, a greater reduction in the growth of bacteria treated simultaneously with ciprofloxacin and carvedilol in comparison with the sum of their separate antibacterial activities indicated supra-additive synergy.

### 2.2. Evaluation of the Mechanism of the Synergistic Action of Ciprofloxacin and Carvedilol

**Figure 2:**
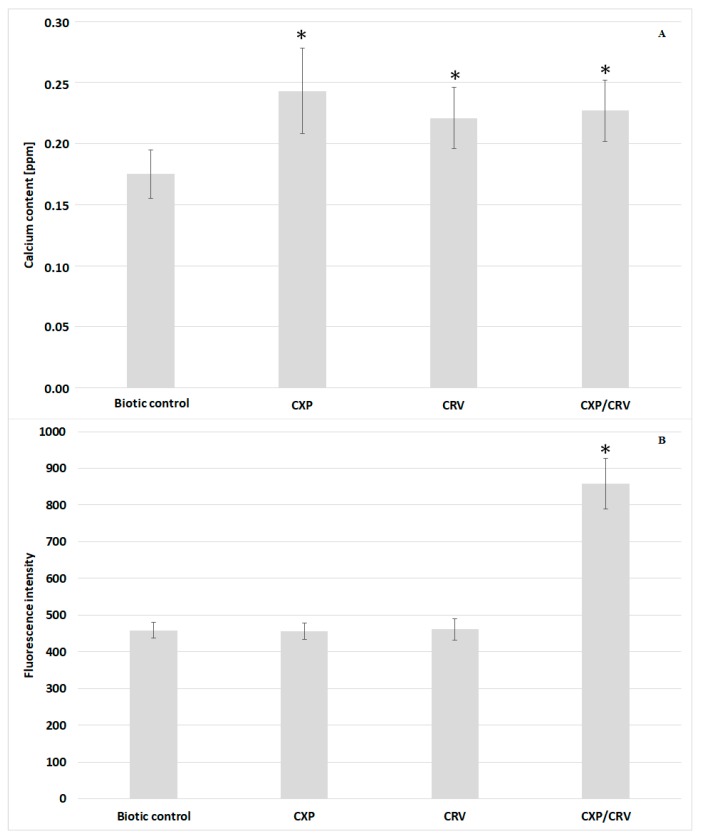
Calcium content in *S. aureus* ATCC 29213 incubated with and without ciprofloxacin (0.1 mg/L) and/or carvedilol (6.25 mg/L). The total calcium level in bacterial cells was measured by AAS (**A**) and the cytosolic free calcium concentration was measured using the fluorescent indicator Fluo 3-AM (**B**). Data (mean ± SD) were analyzed using the one-way and two-way analysis of variance (ANOVA) with * *p* < 0.05.

**Figure 3:**
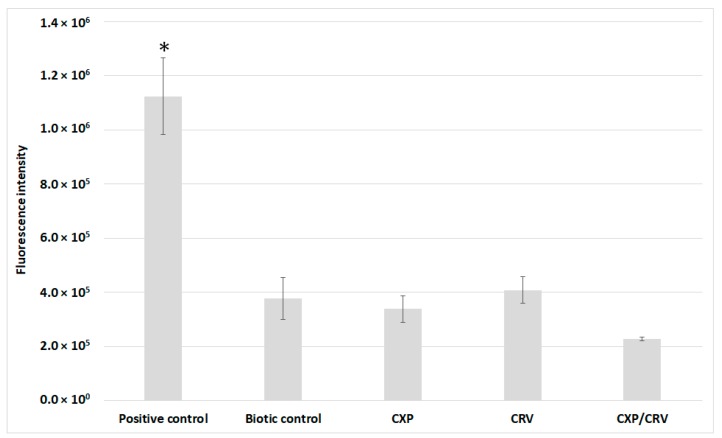
Reactive oxygen species (ROS) production in *S. aureus* ATCC 29213 cells treated and untreated with ciprofloxacin (0.1 mg/L) and/or carvedilol (6.25 mg/L). Data (mean ± SD) were analyzed using the one-way and two-way analysis of variance (ANOVA) with * *p* < 0.05.

Studies on the mechanism of synergistic action of ciprofloxacin and carvedilol were carried out against the most sensitive *S. aureus* ATCC 29213 strain. The total calcium content was measured after mineralization of *S. aureus* cells treated and untreated with ciprofloxacin and/or carvedilol. The obtained results did not show significant differences in the total calcium content in control cells compared to the bacteria incubated with the tested compounds (Figure 2A). Furthermore, the content of free Ca^2+^ was determined using Fluo 3-AM dye, which shows an increase in fluorescence after binding with free calcium ions. The changes in the fluorescence intensity in *S. aureus* cells incubated with and without ciprofloxacin and/or carvedilol have been summarized in Figure 2B. In the cultures supplemented with ciprofloxacin or carvedilol, the measured level of fluorescence intensity was the same as in the control samples. However, coadministration of ciprofloxacin and carvedilol caused an approximately 2-fold increase in Fluo 3-AM fluorescence. The obtained results indicate a significant growth in the content of free calcium ions in bacterial cells simultaneously treated with ciprofloxacin and carvedilol. The changes in the calcium level in bacterial cells could have been related to the induction of the cell death pathway through reactive oxygen species (ROS); therefore, ROS production in *S. aureus* ATCC 29213 cells treated and untreated with ciprofloxacin and/or carvedilol was evaluated (Figure 3). Bacterial cells incubated with H_2_O_2_ were used as a positive control. The fluorescence intensity of the CM-H2DCFDA reagent was approximately 3-fold higher in the positive control than in the other samples. The fluorescence intensity of bacterial cells in the biotic control and samples supplemented with ciprofloxacin and carvedilol reached an average value of 3.75 × 10^5^, and in *S. aureus* cultures simultaneously treated with the antibiotic and the β-blocker, the value was 2.28 × 10^5^. The results did not confirm the induction of ROS formation in response to the simultaneous administration of the tested compounds as a mechanism of killing bacteria. 

**Figure 4:**
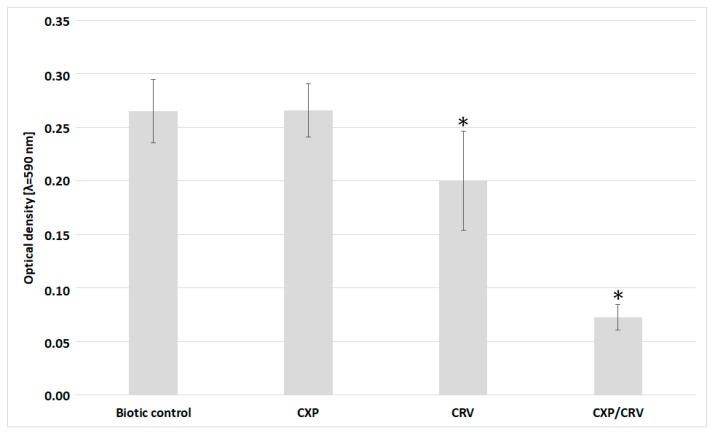
Biofilm formation in *S. aureus* ATCC 29213 cultures treated and untreated with ciprofloxacin (0.1 mg/L) and/or carvedilol (6.25 mg/L). Data (mean ± SD) were analyzed using the one-way and two-way analysis of variance (ANOVA) with * *p* < 0.05.

In the next step, the capacity of *S. aureus* ATCC 29213 cells to form biofilm was evaluated by the measurement of optical density after crystal violet staining (Figure 4). In the biotic controls and cultures treated with ciprofloxacin, the optical density reached a value of approximately 0.26, whereas the addition of carvedilol reduced it to 0.19. The optical density for bacteria simultaneously treated with ciprofloxacin and carvedilol was 0.07. The statistically significant differences have been observed between biotic control and cultures supplemented with carvedilol alone or cultures supplemented simultaneously with ciprofloxacin and carvedilol. The p-values in the ANOVA analyses carried out at the significance level of 0.05 are 0.00042 and 0.000003, respectively. In the case of *S. aureus* cultures incubated with the addition of ciprofloxacin alone, the *p*-value is 0.475937; therefore, the result is not statistically significant. The obtained results show that *S. aureus* cells had a moderate to high capability to form biofilm structures both in the biotic control and in the presence of the tested compound that was separately added. In the case of the bacterial culture supplemented with ciprofloxacin and carvedilol, the ability of *S. aureus* to form biofilms was abolished. 

**Figure 5:**
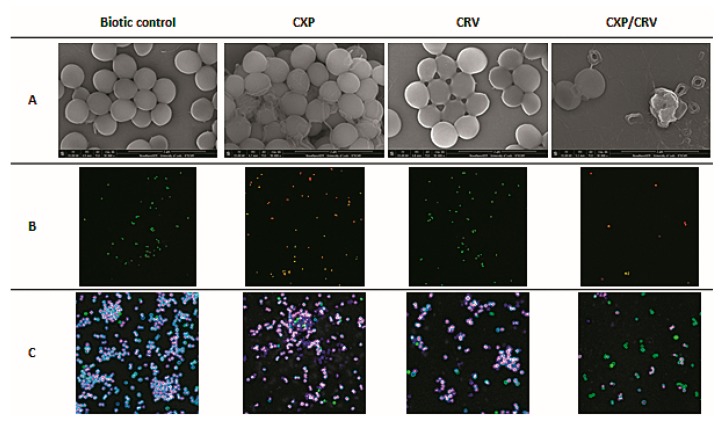
SEM (**A**) and confocal images (**B**,**C**) of *S. aureus* ATCC 29213 incubated with and without the addition of ciprofloxacin (0.1 mg/L) and/or carvedilol (6.25 mg/L). Bacterial cells were stained with the LIVE/DEADTM BacLightTM Bacterial Viability Kit (**B**) and the Alexa Fluor 488/Syto 59 solution (**C**).

The influence of ciprofloxacin and carvedilol on the permeability of the bacterial cell membrane was also examined (Figure 5B). A significant increase of *S. aureus* membrane permeability was observed in bacteria only incubated with ciprofloxacin or simultaneously with the antibiotic and the β-blocker compared to the biotic control. In cultures supplemented with carvedilol alone, bacterial cells did not show changes in membrane permeability.

The morphology of *S. aureus* cells treated with ciprofloxacin and carvedilol was visualized using scanning electron microscopy (Figure 5A). Bacteria only incubated with ciprofloxacin formed clusters embedded in specific network-like structures. These structures were synthesized to a much smaller extent around the cells that were treated and untreated with only carvedilol. In the case of simultaneous administration of the antibiotic and the β-blocker, *S. aureus* cells were wrinkled or completely damaged. The results confirm that the clumping of *S. aureus* cells and the formation of cellular aggregates in planktonic cultures. 

Extracellular DNA (eDNA)-mediated *S. aureus* cell aggregation was assessed according to the method of Grande et al. (2014). The Alexa Fluor 488 was used to visualize staphylococcal cells (green fluorescence) and Syto 59 was used to stain the DNA (red and blue fluorescence). The images obtained from the three channels were overlaid and revealed bacterial cells colored with a pink and blue shadow of eDNA around them (Figure 5C). The bluest shadows were observed in the cultures incubated with the addition of ciprofloxacin and slightly less was observed around the cells treated with carvedilol. The analyzed images indicated that eDNA was engaged in the formation of *S. aureus* aggregates. In the samples simultaneously supplemented with the antibiotic and the β-blocker, a significant number of bacterial cells did not stain with Syto 59. Overall, the green cells are supposed to represent lysed cells.

## 3. Discussion

Literature data on the antimicrobial properties of carbazole derivatives, such as carvedilol, are still limited. To date, the moderate antibacterial potential of carbazolyl glyoxamides or β-carboline chalcones against Gram-negative and Gram-positive bacteria has been demonstrated [11,12]. Our previous studies have shown the antimicrobial potential of carvedilol at high concentrations against Gram-positive bacteria [9]. The minimum inhibitory concentrations (MICs) of the β-blocker for *S. aureus* and *S. pyogenes* were 40 and 45 mg/L, respectively. Moreover, changes in the fatty acid profile and an increase in membrane permeability were detected both in Gram-negative and Gram-positive bacteria [9]. In the light of these reports, the presented results regarding the synergy of ciprofloxacin and carvedilol seem valuable as a potential new alternative strategy of infectious disease treatment. However, further in vivo studies and an explanation of the mechanism of synergistic interaction of the tested compounds are needed. 

Calcium is one of the most important cell regulators in eukaryotes, but its role in prokaryotic cells is not yet well understood. However, it was found that bacterial cells maintain calcium homeostasis due to membrane ion channels, transporters, and calcium-binding proteins. Calcium ions are probably engaged in cell structure maintenance, the motility of bacterial cells, the expression of genes, and cell division and their differentiation. It has also been shown that changes in the level of free Ca^2+^ ions can be a signal mechanism [13]. In the case of carvedilol and ciprofloxacin, the calcium channel blocking activity has been stated [6,14]. Therefore, the studies of the mechanism of the synergistic action of ciprofloxacin and carvedilol included the analysis of calcium content in *S. aureus* cells untreated and treated alone and in combination with the tested compound. Although both tested compounds show calcium channel blocker activity, an increase in the free Ca^2+^ level was observed in *S. aureus* cells incubated simultaneously with the antibiotic and the β-blocker, while total calcium content was similar in all tested samples. The observed effect might be a result of the interaction of ciprofloxacin with carvedilol and as a consequence formation of a new complex with a stronger antimicrobial effect than the drugs using separately.

The changes in the calcium level in eukaryotic cells are related to the production of reactive oxygen species (ROS) and the initiation of the pathways of cell death such as necrosis or apoptosis [15]. However, in the present study, induction of reactive oxygen species formation was not demonstrated after the treatment of *S. aureus* cells with ciprofloxacin and carvedilol. Therefore, no correlation was found between the increase in calcium ion concentration and the induction of ROS synthesis.

The enhancement of the antimicrobial potential of ciprofloxacin by carvedilol may have been the result of the release of a certain pool of bound calcium. In our previous studies, the modifications of the profile of fatty acids of *S. aureus* cells incubated with carvedilol were shown. These results were also correlated with changes in bacterial membrane permeability [9]. Thus, the release of calcium into the cytosol seems to be possible as a result of destabilization of the cell envelopes. However, in this study, an increase in bacterial membrane permeability was demonstrated in all samples incubated with ciprofloxacin.

Calcium ions can be also engaged in the aggregation of bacterial cells and consequently biofilm formation [16,17]. Pathogens, such as *S. aureus*, may possess an ability to form biofilm structures on abiotic and biotic surfaces that can protect bacteria against the response of the host immune system and against the action of antibacterial agents [18]. The obtained results showed the reduction of *S. aureus* biofilm formation after the simultaneous treatment with ciprofloxacin and carvedilol, which was correlated with an increase in the level of free Ca^2+^ inside the bacterial cells. Moreover, SEM analysis demonstrated formation of specific cell clusters in samples incubated with the antibiotic alone. Watters et al. [19] also showed the ability of *S. aureus* to form similar network-like structures in biofilms. Habber et al. [16] detected cellular aggregates formed in *S. aureus* cultures whose matrix was built of polysaccharide intercellular adhesin (PIA). Bacterial aggregates may contain matrix components such as surface proteins, polysaccharides, or extracellular DNA (eDNA). Protein A-, fibrinogen-, and fibronectin-binding proteins are involved in staphylococcal biofilm formation at an early stage of the process. The next important component of the biofilm matrix can be extracellular polysaccharides (EPSs) such as poly-*N*-acetyl-β-(1,6)-glucosamine. Extracellular DNA, which is a key factor for early biofilm formation, is released during controlled bacterial cell lysis [16]. In addition, the protective role of bacterial aggregates and their contribution to increasing the tolerance of pathogens to antibiotics was indicated [20]. Therefore, the contribution of eDNA to the formation of aggregates of *S. aureus* cells treated with ciprofloxacin and carvedilol was also assessed. The participation of eDNA in the formation of bacterial aggregates was confirmed in samples incubated separately with the addition of the antibiotic and carvedilol. Moreover, microscopic analysis of *S. aureus* cells incubated without and with the addition of ciprofloxacin and/or carvedilol revealed significantly less bacterial cell clusters formed in cultures supplemented simultaneously with both tested compounds. The obtained results suggest a possible negative impact of both tested compounds added simultaneously to *S. aureus* cultures on the ability of bacteria to form cell clusters, which might have prevented *S. aureus* cell growth. 

Although ciprofloxacin causes many side effects, it is still one of the most commonly used fluoroquinolone antibiotics. Application of ciprofloxacin commonly leads to the reactions of the central nervous system and the digestive system including vomiting, nausea, diarrhea, sleeplessness, dizziness, or headache. Moreover, tendon rupture, muscle weakness, and joint inflammation were observed in patients treated with ciprofloxacin [21,22]. The side effects of the tested β-blocker are similar to those caused by ciprofloxacin, such as nausea, vomiting, and diarrhea. Edema, bradycardia, hypotension, and blurred vision are also frequently noted in patients cured with the antibiotic [23]. The occurrence of similar side effects in therapy with both compounds may be a problem in clinical use; therefore, further in vivo experiments are required. 

## 4. Materials and Methods 

### 4.1. Standards and Reagents

Carvedilol (PHR1265) and ciprofloxacin (17850) were purchased from Sigma-Aldrich (Poznań, Poland). Cation-adjusted Mueller Hinton II Broth (MHB) was obtained from Becton Dickinson (Warsaw, Poland). Fluo 3-AM and a LIVE/DEADTM BacLightTM Bacterial Viability Kit were purchased from Thermo Fisher Scientific (Warsaw, Poland). Glutaraldehyde and osmium tetroxide were obtained from Agar Scientific (Stansted, UK). All of the other reagents with a high analytical purity grade were obtained from Sigma-Aldrich (Poznań, Poland). 

### 4.2. Carvedilol and Ciprofloxacin Susceptibility Testing

The antibacterial potential of ciprofloxacin (CXP) and carvedilol (CRV) was tested by the microdilution method in Mueller Hinton Broth (MHB) according to CLSI and EUCAST guidelines. Susceptibility testing assays were conducted on *S. aureus* ATCC 6538, *S. aureus* ATCC 29213, and *S. aureus* ATCC 43300 in 96-well cell culture plates. The antibacterial activity of CXP and CRV were assessed over concentration ranges of 0.025–0.6 mg/L and 1.56–70 mg/L, respectively. The stock solutions of CRV and CXP at the concentration 25 mg/mL were prepared in dimethyl sulfoxide and deionized water with the addition of 0.1 M HCl, respectively. The CRV and CXP solutions were then diluted in MHB to achieve the final tested concentrations and to minimize the effect of solvents on the bacterial growth. A final bacterial density in each well was 5 × 10^5^ CFU/mL. Samples containing CRV and/or CXP as well as adequate abiotic and biotic controls were incubated for 24 h at 37 °C. The optical density of all tested combinations was measured spectrophotometrically (λ = 630 nm) using a MultiskanTM FC Microplate Photometer (Thermo Fisher Scientific). The results of bacterial susceptibility are shown as a percentage of the control group (*n* = 4) from four separate experiments. 

The minimum inhibitory concentration (MIC) was designed as the lowest concentration of ciprofloxacin or carvedilol that inhibited the growth of 99% microorganisms. The fractional inhibitory concentration (FIC) of ciprofloxacin or carvedilol was calculated from the formulas:

$$FIC_{CXP/CRV}=\frac{{\mathrm{MIC}}_{CXP/CRV\;in\;combination}}{{\mathrm{MIC}}_{CXP/CRV\;alone}}$$

The fractional inhibitory concentration index (FICI) of the tested drugs was calculated from the formula:

$$FICI=\sum^{\text{​}}FIC=\;\frac{{\mathrm{MIC}}_{CXP\;in\;combination}}{{\mathrm{MIC}}_{CXP\;alone}}+\;\frac{{\mathrm{MIC}}_{CRV\;in\;combination}}{{\mathrm{MIC}}_{CRV\;alone}}$$

FICI ≤ 0.5 was defined as synergy [24].

### 4.3. Assay of Calcium Content in the Bacterial Cells

The calcium content in *S. aureus* cells incubated with and without the addition of CRV and/or CXP was measured using flow cytometry and atomic absorption spectroscopy. The bacterial cells were centrifuged at 10,000 rpm for 4 min and washed thrice with phosphate buffered saline (PBS). A part of the *S. aureus* cells was suspended in a Fluo 3-AM solution prepared in PBS and incubated at 37 °C for 60 min in the dark. Then, bacterial cells were centrifuged at 10,000 rpm for 4 min and washed thrice with PBS. Finally, bacteria were suspended in 500 µL PBS and analyzed on an LSR II (Becton Dickinson, San Jose, CA, USA) instrument by acquiring 30,000 events per sample. Untreated *S. aureus* cells were used as the control. The fluorescence of a Fluo 3-AM stain was excited by an argon-ion laser at 488 nm and collected at 530 nm. All experiments were prepared in triplicate in two independent tests. The calcium ion content in *S. aureus* cells was expressed as the median of the histogram fluorescence intensity of Fluo 3-AM.

The second part of the bacterial cells was washed away from the MHB medium and filled with 5 mL of nitric acid and mineralized at 148 °C for 4 h. Each sample was diluted five times in deionized water and the calcium concentration was measured using a Varian SpectrAA 300 Flame Atomic Absorption Spectrophotometer (Varian, Palo Alto, CA, USA). 

The experiments were carried out in four independent repetitions.

### 4.4. ROS Production Measurement

ROS production was measured in *S. aureus* cells untreated and treated with CXP and/or CRV for 30 min using the CM-H2DCFDA reagent according to the producer’s protocol. Bacterial cells incubated with H_2_O_2_ at a concentration of 100 µM for 30 min were used as a positive control. After 30 min incubation with the tested compounds, the *S. aureus* cells were centrifuged at 10,000 rpm for 4 min and washed thrice with phosphate buffered saline (PBS). Then, the bacterial cells were suspended in a CM-H2DCFDA solution at a concentration of 5 µM and incubated at 37 °C for 30 min in the dark. Next, the cells were again centrifuged at 10,000 rpm for 4 min and washed with PBS. The fluorescence intensity was measured at the excitation/emission (ex/em) maxima, 495/520 nm, using a SpectraMax i3x Multi-Mode Microplate Reader (Molecular Devices Ltd, Wokingham Berkshire/UK). The experiment was carried out in three independent repetitions.

### 4.5. Biofilm Formation Measurement

Biofilm formation was evaluated in 96-well cell culture plates similarly to CRV and CXP susceptibility testing. After 24 h of incubation, the bacterial suspensions with and without CRV and/or CXP were removed from the wells. Then, each well was washed twice with 0.9% NaCl and stained with a 0.1% crystal violet solution prepared in 0.9% NaCl. Next, the wells were washed with deionized water and dried at 37 °C for 30 min. Finally, the wells were washed with 80% ethanol and deionized water. The absorbance was measured at 590 nm. The experiment was carried out in four independent repetitions. 

### 4.6. Confocal Microscopy 

Cell visualization was conducted in the Laboratory of Microscopic Imaging and Specialized Biological Techniques (Faculty of Biology and Environmental Protection, University of Lodz) using a Leica TCS SP8 microscope equipped with plan achromatic objectives (Leica) and with magnifications of 63× (Water immersion) and 100× (Oil immersion).

*S. aureus* cells were stained using the LIVE/DEADTM BacLightTM Bacterial Viability Kit according to the producer’s protocol. The bacterial cells treated and untreated with CXP and/or CRV were centrifuged at 10,000 rpm for 4 min and washed thrice with PBS. Then, the cells were suspended in 100 µL PBS with the addition of 1 µL of the Syto 9 and propidium iodide mixture (*v/v*:1/1). Next, the bacterial suspensions were vortexed and incubated at 37 °C for 15 min in the dark. Finally, the fluorescence of Syto 9 and propidium iodide was measured at the excitation/emission (ex/em) maxima of 480/500 nm and 490/635 nm, respectively. 

The bacterial cells were also stained with the Alexa Fluor 488 and the Syto 59 dyes according to the producer’s protocol. *S. aureus* cells were separated from the growth medium and washed thrice with PBS and then suspended in 150 µL Alexa Fluor 488 and Syto 59 solution at the concentrations of 0.3 and 0.02 µM, respectively. The bacterial cells were incubated at 37 °C for 30 min in the dark and then centrifuged at 10,000 rpm for 4 min and washed thrice with PBS. Next, the fluorescence of the Alexa Fluor 488 was detected at the (ex/em) maxima of 495/519 nm and 490/635 nm. Finally, the Syto 59 fluorescence was visualized at the (ex/em) maxima of 622/640-660 nm and 622/660-720 nm. The experiments were carried out in three independent repetitions. 

### 4.7. Scanning Electron Microscopy

*S. aureus* cultures incubated with and without CXP, CRV, or CXP/CRV were centrifuged at 10,000 rpm for 4 min and washed thrice with PBS. Next, the bacterial cells were suspended in a glutaraldehyde solution and incubated for 16 h. After incubation, *S. aureus* cells were again centrifuged at 10,000 rpm for 4 min and washed thrice with PBS. The obtained precipitates were suspended in an osmium tetroxide solution and incubated for 20 min. Next, the bacterial suspensions were centrifuged and washed in PBS and then dehydrated in a series of ethanol solutions (25%, 50%, 75%, 90%, and 100%) for 15 min each. *S. aureus* cells were spread on a silicon wafer, dried at room temperature and sputtered with an Au layer at a thickness of 2 nm. SEM images of the treated and untreated *S. aureus* cells were performed using a Nova NanoSEM™ scanning electron microscope. The images were made in an immersion mode using a through lens detector (TLD) at a typical magnification of 50,000×. The experiment was carried out in three independent repetitions.

### 4.8. Statistical Analysis

All experiments were at least performed in triplicate. The obtained values were expressed as the means with standard deviation (SD). The one-way and two-way analyses of variance (ANOVA) with *p* < 0.05 were considered statistically significant calculations using Excel, Microsoft ^®^ Office 2016 (Microsoft Corporation, Redmond, WA, USA).

## 5. Conclusions

In conclusion, it should be emphasized that carvedilol enhances the antimicrobial action of ciprofloxacin. This effect is correlated with a disturbance of calcium homeostasis in *S. aureus* cells, morphological changes to the cells, and a reduction in the ability to form bacterial aggregates.

## Figures and Tables

**Table 1:** In vitro antibacterial activity of ciprofloxacin combined with carvedilol against *S. aureus* strains.

Strain	MIC*_CXP_* (mg/L)	MIC*_CRV_* (mg/L)	FIC_CXP_	FIC_CRV_	FICI
ATCC 6538	0.25	40	0.100	0.039	0.139
ATCC 29213	0.40	50	0.063	0.031	0.094
ATCC 43300	0.45	60	0.055	0.026	0.081
